# Tuning VSV-G Expression Improves Baculovirus Integrity, Stability and Mammalian Cell Transduction Efficiency

**DOI:** 10.3390/v16091475

**Published:** 2024-09-17

**Authors:** Martina Mattioli, Renata A. Raele, Gunjan Gautam, Ufuk Borucu, Christiane Schaffitzel, Francesco Aulicino, Imre Berger

**Affiliations:** 1 School of Biochemistry, University of Bristol, 1 Tankard’s Close, Bristol BS8 1TD, UK; martina.mattioli.2020@bristol.ac.uk (M.M.); renata.raele@bristol.ac.uk (R.A.R.); gunjan.gautam@bristol.ac.uk (G.G.); christiane.berger-schaffitzel@bristol.ac.uk (C.S.); 2 GW4 Cryo-EM Facility, University of Bristol, Life Sciences Building, Bristol BS8 1TQ, UK; ufuk.borucu@bristol.ac.uk; 3 School of Chemistry, University of Bristol, Cantock’s Close, Bristol BS8 1TS, UK; 4 Max Planck Bristol Centre for Minimal Biology, Cantock’s Close, Bristol BS8 1TS, UK

**Keywords:** baculovirus, pseudotyping, VSV-G, AcMNPV, gene delivery, viral vector

## Abstract

Baculoviral vectors (BVs) derived from *Autographa californica* multiple nucleopolyhedrovirus (AcMNPV) are an attractive tool for multigene delivery in mammalian cells, which is particularly relevant for CRISPR technologies. Most applications in mammalian cells rely on BVs that are pseudotyped with vesicular stomatitis virus G-protein (VSV-G) to promote efficient endosomal release. VSV-G expression typically occurs under the control of the hyperactive polH promoter. In this study, we demonstrate that polH-driven VSV-G expression results in BVs characterised by reduced stability, impaired morphology, and VSV-G induced toxicity at high multiplicities of transduction (MOTs) in target mammalian cells. To overcome these drawbacks, we explored five alternative viral promoters with the aim of optimising VSV-G levels displayed on the pseudotyped BVs. We report that Orf-13 and Orf-81 promoters reduce VSV-G expression to less than 5% of polH, rescuing BV morphology and stability. In a panel of human cell lines, we elucidate that BVs with reduced VSV-G support efficient gene delivery and CRISPR-mediated gene editing, at levels comparable to those obtained previously with polH VSV-G-pseudotyped BVs (polH VSV-G BV). These results demonstrate that VSV-G hyperexpression is not required for efficient transduction of mammalian cells. By contrast, reduced VSV-G expression confers similar transduction dynamics while substantially improving BV integrity, structure, and stability.

## 1. Introduction

Baculoviral vectors (BVs) derived from *Autographa californica* multiple nucleopolyhedrovirus (AcMNPV) are insect-specific vectors found to efficiently transduce a wide range of human cell lines in which they neither replicate nor integrate, rendering them safe [1,2,3]. Baculoviruses in nature range widely in genomic size, from 120 to 180 kb [4,5]. BVs can easily accommodate large synthetic DNA cargoes (50–100 kb) due to their expandable rod-shaped capsid, with currently unknown upper limit. We and others have extensively exploited this feature for multiprotein complex production in insect cells [6,7,8,9] and multigene delivery to mammalian cells [3]. 

By contrast, the rigid capsid architectures of commonly employed viral vectors impose a hard cap on their genetic cargo capacity, which is limited to 4.5 kb for adeno-associated vectors (AAVs), 8.5 kb for lentiviruses (LV) and 36 kb for third-generation high-capacity adenoviruses (HC-AdVs) [2], which currently dominate the field. This represents a major limitation, particularly in the context of CRISPR-based genome editing. Indeed, more sophisticated editing toolkits including gene replacement [10], prime editing [11] and PASTE [12], cannot typically be delivered using a single vector, contributing to decreased gene editing success rates.

We and others have demonstrated that BVs can deliver different CRISPR technologies to a range of cultured human cells [3,13,14], enabling efficient, large DNA cargo integration and multiplexed prime editing using a single all-in-one baculovirus [15]. More recently we have also demonstrated that, alongside their DNA cargo, BVs can simultaneously deliver Cas9 protein, resulting in efficient gene editing in the absence of detectable off-target effects [16]. 

The major AcMNPV glycoprotein, GP64, mediates BV cell entry by binding heparan sulphate on target mammalian cells [17,18], but in early endosomes in the mammalian host, the pH is often above the pH (<5) at which GP64 transitions to a post-fusion state. This results in incomplete endosomal escape of BVs in mammalian cells, which in turns reduces transduction efficiencies, sometimes severely [19]. For this reason, since its early inception, BV transduction in mammalian cells has exploited pseudotyping with vesicular stomatitis virus glycoprotein (VSV-G) [20]. VSV-G binds to low-density lipoprotein receptor (LDL-R) [21] which is abundantly expressed on the target mammalian cells and, at the same time, transitions from the pre-fusion to post-fusion state at pH 6.0–6.5, triggering efficient early-endosomal release [22].

BV pseudotyping is typically achieved by including a cassette in which VSV-G is placed under the control of the hyperactive very late baculoviral polyhedrin promoter (polH) either as a plasmid module (BacMam 2.0, ThermoFisher), embedded in the viral genome (MultiBacMam, GenevaBiotech) or in packaging insect cell lines under the control of a polH [23] or a polH-chimeric promoter [24]. In all instances, BVs equipped with VSV-G display broader mammalian cell tropism and superior transduction efficiencies as compared to non-pseudotyped BVs [3,15,20,25]. While representing a successful strategy, polH-driven expression nonetheless triggers massive incorporation of VSV-G on the baculovirions, significantly outcompeting GP64 abundance as evidenced by mass spectrometry [16]. VSV-G hyperexpression is also linked to increased cytotoxicity in mammalian cells [26]. Moreover, VSV-G can interfere with BV nanostructures by altering the envelope morphology [20]. In addition, we noticed that preparing stocks of polH VSV-G BVs by concentration results in significantly reduced infectious titres as compared to non-pseudotyped BVs. In the past, BVs pseudotyped with truncated VSV-G (VSV-GED) proteins have been engineered to address some of these issues [26], although it was later found that transduction efficiencies otherwise conferred by full-length VSV-G are restored only to a negligible extent by using VSV-GED [27]. 

In the present study, we aimed to resolve these issues by testing alternative viral promoters to drive VSV-G expression during baculovirus packaging in insect cells. Our objective was to identify optimal VSV-G expression levels for maintaining the beneficial, high levels of transduction efficiencies of the pseudotyped BV, while at the same time reducing or eliminating cytotoxicity and viral stability defects. By testing six viral promoters (including polH) and systematically comparing the resulting VSV-G-enveloped BVs with a reference wild-type (non-VSV-G) BV, we identified Orf-13 and Orf-81 as alternative promoters to drive VSV-G expression. Although Orf-13 and Orf-18 promoters lead to less than 5% VSV-G incorporation on budded virions as compared to polH, the resulting viruses preserved high transduction efficiencies in target mammalian cells. Moreover, we found that the reduced VSV-G expression levels rescued BV structural defects caused by polH-driven VSV-G hyperexpression and improved the stability of concentrated BV stocks. Furthermore, BVs pseudotyped with reduced VSV-G levels displayed lower cytotoxicity in target human cells, including primary cells. Finally, these reduced VSV-G BVs efficiently delivered multigene CRISPR constructs, maintaining transduction and gene editing efficiencies overall comparable to the original polH VSV-G BVs. 

Taken together, our results demonstrate that finetuning of the viral promoter driving glycoprotein expression is instrumental for optimal pseudotyping and the development of safer and stable BVs for gene delivery and editing in mammalian cells.

## 2. Materials and Methods

### 2.1. Plasmid Design and Cloning

Alternative viral promoters were synthesised by GenScript and cloned into pIDC VSV-G vector using BamHI/PmeI restriction sites. pIDC VSV-G and pIDC-derived vectors were propagated in Pir+ *E. coli* with chloramphenicol (30 mg/mL). The pIDC backbone (without VSV-G) can be accessed on GenBank (LQ942132.1). The resulting pIDC with variable promoters were individually assembled with pACEMam1 EGFP WPRE (modified from pACEMam1) [3] via Cre-mediated recombination as previously described [3,6,7,15]. Briefly, acceptor and donor plasmids were mixed in equimolar amounts (total DNA amount was 500 ng) with 0.5 μL Cre recombinase (NEB, Ipswich, MA, USA # M0298M), 1 μL Cre-recombinase reaction buffer (provided with Cre recombinase) and ddH_2_O to 10 μL. Reactions were incubated at 37 °C for 1 h and transformed in homemade electrocompetent Top10 *E. coli* under gentamycin (10 mg/mL) and chloramphenicol (30 mg/mL) antibiotic selection. Individual colonies were screened by restriction digestion to confirm the absence of acceptor/donor duplications. The digestion patterns were compared to in silico assembled Cre reactions performed with CRE-ACEMBLER software [28] before proceeding to the next steps. For the production of all-in-one CRISPR BV, the same procedure was applied by using MultiMate HITI-2c ACTB (Addgene, Watertown, NY, USA, plasmid#206267) [15] as the acceptor partner. All plasmid sequences are available in Supplementary Table S2.

### 2.2. Baculovirus Amplification

Assembled plasmids were transformed in chemically competent DH10-EmbacY *E. coli*. Plasmids were integrated into the baculovirus genomes by Tn7 transposition following standard protocols. After overnight recovery in LB media, bacteria were plated, and positive colonies selected by blue–white screening. Bacmid extraction was performed using alkaline lysis/ethanol precipitation as previously described [6,7,29]. 

Transfection of the purified bacmid into Sf21 cells as well as first (V0) and second (V1) generation viral amplification and harvest were performed as previously described. Third-generation viruses (V2) were amplified by infecting 50 mL of cells at 0.8 × 10^6^ cells/mL with 1 mL of V1. V2 supernatants were clarified by centrifugation at 3500× *g* for 5 min to remove cell debris and stored at 4 °C. 

BVs viral DNA was extracted and quantified by qPCR as previously described [16] using oligonucleotides targeting gp64 (Forward 5′-CGCTTCACCAACTCTTTGCC-3′) and reverse 5’-AAGAGCTGATCGACCGTTGG-3′). An extended protocol covering baculovirus amplification and quantification using qPCR has been recently published by our group (Aulicino et al. 2024, “Assembly of baculovirus vectors for multiplexed prime editing”, *Methods in Mol. Biology*) [29]. 

### 2.3. Cell Culture

Sf21 insect cell cultures (Thermo Fisher Scientific, Waltham, MA, USA #IPLB-Sf21-AE) were maintained at 0.5–2.0 × 10^6^ cells/mL in ESF 921 culture media (Expression Systems #96-001-01). The cells were kept on a shaker (Thermo Fisher Scientific, Waltham, MA, USA) in 125–250 mL polycarbonate flasks (CORNING, New York, US, #431143, #431144) at 27 °C as previously described [15,16,29]. Mammalian cells were cultured as previously described [16]. Briefly, HEK293T (ATCC #CRL-3216), RPE1-hTERT, (ATCC, Gaithersburg, US #CRL-4000), SH-SY5Y (ATCC#CRL-2266), and HeLa (ATCC #CRM-CCL-2) were cultured in Dulbecco’s Modified Eagle Media (DMEM) (Gibco, Thermo Fisher Scientific, Waltham, MA, USA #41965), supplemented with 10% foetal bovine serum (FBS) (Gibco #A4766801), 10 U/mL penicillin, and 10 µg/mL streptomycin (Gibco #11548876). Primary HUVECs (Merck, Darmstadt, Germany #C-12203) were cultured in Endothelial cell growth medium kit (Merck #C-22110) supplemented with 10 U/mL penicillin and 10 µg/mL streptomycin (Gibco #11548876). A549 cells (Merck 86012804–1VL) were cultured in Gibco Ham’s F-12K (Kaighn’s) Medium supplemented (Fisher Scientific #11580556) with 10% FBS (Gibco #A4766801), 10 U/mL penicillin, and 10 µg/mL streptomycin (Gibco #11548876).

### 2.4. Mammalian Cells Transduction and Fluorescent Titration Assay

One day before transduction, mammalian cells were seeded at 2 × 10^4^ cells/well in 96-well plates in 100 μL of complete medium. The next day, the viral supernatants were diluted with DPBS pH 7.4 to obtain the desired gc/cell ratios, and 25 μL of each viral dilution was added to each well. The plates were spinoculated at 300× *g* for 30 min at 27 °C, followed by overnight incubation with viral supernatants. For titration experiments on HEK293T, the cells were analysed via flow cytometry at 24 h post-transduction for EGFP expression. For all the other experiments, 24 h post-transduction, the media containing the viral supernatants were aspirated and replaced with fresh medium. The cells were analysed by flow cytometry (to evaluate transduction or gene editing efficiencies) or a plate reader (transgene expression and viability) at 48 h post-transduction. 

### 2.5. Plate Reader Measurements of EYFP, EGFP, and alamarBlue 

For analysis of EYFP expression, Sf21 cells cultured in 6-well plates were analysed 72 h post bacmid transfection or transduction, virus-containing supernatants were aspirated at 72 h, and cells were overlaid with 2 mL DPBS pH 7.4 to reduce autofluorescence caused by ESF 921 medium. To cover the whole well area, 25-tiled measurements were carried out for each well/transfection using a Biotek Synergy H1 (Agilent, Santa Clara, CA, USA).

For analysis of EGFP expression in HEK293T, A549, HeLa, and HUVEC, cells cultured in 96-well plates were analysed at 48 h post-transduction in complete media using a Biotek Synergy H1 (Agilent). For analysis of cell viability, cell media were replaced with 10% alamarBlue reagent (ThermoFisher #Dal1025) following the manufacturer’s recommendations. 4 h after alamarBlue addition, the cells were analysed using a Biotek Synergy H1 (Agilent) to monitor the conversion of resazurin (non-fluorescent) to resafurin (fluorescent). Relative fluorescent units (RFUs) were scaled to RFUs obtained in untransduced control cells to obtain the percentage of viable cells at 48 h post-transduction.

### 2.6. Western Blot 

Protein extracts from 1 × 10^6^ Sf21 insect cells were prepared by extraction in RIPA buffer (ThermoFisher # 89900) following the manufacturer’s recommendations. Viral protein extracts were obtained by concentration of titre-normalised viral supernatants by centrifugation at 47,500× *g* for 1 h at 4 °C using a Sorvall Lynx 6000 centrifuge (Thermo Scientific). Viral pellets were resuspended in RIPA buffer (1:50 of the original supernatant volume). The protein extracts were mixed 3:1 with 4× Laemmli buffer, followed by incubation at 95 °C for 5 min. Electrophoresis was performed on 3–8% tris-acetate gels (Novex, Thermo Fischer Scientific) using 1× Tris Acetate Buffer (Novex) for 60–80 min at 200 V. The PageRuler Plus Protein Standard (Thermo Scientific) was used as a molecular weight marker. For Coomassie labelling experiments, membranes were stained with Coomassie Fluor (ThermoFisher# C33250) following the manufacturer’s recommendations.

For western blot experiments, the gels were transferred to 0.22 µm nitrocellulose or PVDF membranes (BioRad, Hercules, CA, USA) using the iBlot2 dry blotting system (Invitrogen). The membranes were blocked for 1 h at room temperature using 5% skim milk powder in PBS (pH 7.4) with 0.1% Tween20 (PBS-T) and then incubated with primary antibodies against GP64 (Santa Cruz Biotechnology, Dallas, TX, USA #sc-65499), VSV-G (antibodies.com, Cambridge, UK #A121626), or ACTB-HRP (abcam, Cambridge, UK #ab49900) overnight at 4 °C with gentle agitation following the manufacturer’s recommendations. The membranes were washed for 3 × 5 min in PBS-T, before incubation with HRP-conjugated secondary antibodies (anti-mouse HRP-conjugated IgG secondary (Sigma, St. Louis, MO, USA #A5906) or anti-goat HRP-conjugated IgG secondary (Santa Cruz Biotechnology #sc-2020) at room temperature for 1 h. After washing, signals were imaged on a gel imaging system (Syngene) after developing the membrane with SuperSignal West Pico PLUS chemiluminescent substrate (Thermo Scientific). For the conjugated ACTB-HRP antibody, the membranes were imaged after incubation with the primary antibody.

### 2.7. Cryo-EM

For cryo-EM experiments, titre-normalised viral supernatants were concentrated 10 times in ES921 medium using high-speed centrifugation (47,500× *g*, 1 h at 4 °C). A total of 5 μL of each specimen was applied to freshly glow-discharged (1 min. at 5 mA) Lacey carbon films on 200-mesh copper grids and was vitrified using Vitrobot MarkIV (Thermo Fisher Scientific) at 100% humidity and 4 °C with a wait time of 1 s. Afterwards, the grids were blotted for 2 s and plunged frozen into a liquid ethane–propane mixture. Images were acquired on FEI Talos Arctica transmission electron microscope operated at 200 kV and equipped with a Gatan K2 Summit direct detector and Gatan Quantum GIF energy filter, operated in zero-loss mode with a slit width of 20 eV using TOMO software at 49,000× magnification.

### 2.8. Evaluation of Viral Stability upon Concentration

To evaluate viral stability upon concentration, viral genome copies in clarified supernatants were quantified using qPCR and normalised to 1 × 10^9^ cg/mL in ESF921 media. A total of 40 mL of titre-normalised viral supernatants were then concentrated via high-speed centrifugation at 47,500× *g* for 1 h at 4 °C and resuspended in 40 mL (unconcentrated) or 1 mL (40× concentrated) of cold Dulbecco’s Phosphate-Buffered Saline (DPBS) pH 7.4. Unconcentrated and 40× concentrated viruses were stored at 4 °C in the dark for up to 1 week in the absence of cryo-protectants. Transducing titres were estimated at 24 h or at 1 week post-concentration via fluorescent titration assays in HEK293T cells. To enable a comparison of unconcentrated and 40× concentrated viruses, 40× concentrated viral stocks were diluted to their original concentration immediately before testing. 

### 2.9. Widefield and Confocal Microscopy

Widefield imaging was performed on a Leica DMI6000 inverted epifluorescence microscope. Images were acquired with a Photometrics Prime 95B sCMOS Camera (1200 × 1200 11 µm pixels) using Leica LAS-X acquisition software. Mammalian cells were maintained at 37 °C in an environmental control chamber (Solent), and Sf21 insect cells were imaged at room temperature. For confocal microscopy of live cells, images were acquired with a Leica Sp8 microscope equipped with 405, 458, 476, 488, 496, 514, 561, 594, and 633 nm lasers.

## 3. Results

### 3.1. Tuning the Levels of VSV-G Displayed on Pseudotyped BVs 

Expression cassettes based on the hyperactive viral very late promoters polH and p10 dominate heterologous protein production in baculovirus-infected insect cells as they often confer very high protein yields [30]. Although not widely adopted, alternative viral promoters can effectively be used to dose recombinant protein expression dynamics and levels [30,31]. To reduce VSV-G occupancy on budded virions, we choose five viral promoters, attempting to reproduce a balanced range of expression levels from low to high based on transcriptomic data [32]. We selected promoters of the major baculoviral envelope glycoprotein (GP64) [31], the major nucleocapsid protein (VP39) [31,33], a late viral RNA-polymerase subunit (LEF8) [34], a protein involved in nucleocapsid nuclear egress (ORF-13) [35], and a putative viral disulphide isomerase (ORF-13) [36,37]. Promoter sequences and positions are listed in Table S1.

**Figure 1:**
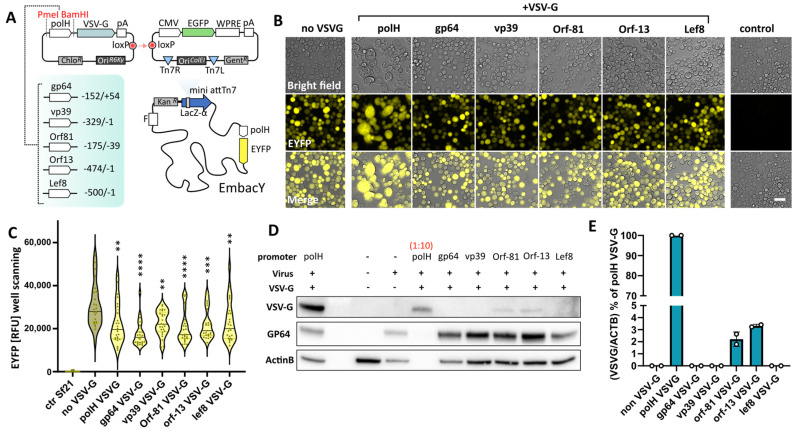
Baculovirus VSV-G pseudotyping using alternative viral promoters. (**A**) Schematic representation of the cloning strategy implemented to generate BVs encoding CMV EGFP (mammalian cells transduction marker), polH EYFP (viral amplification and protein expression marker in insect cells), and VSV-G expression under alternative viral promoters (pseudotyping module). (**B**–**E**) Characterisation of Sf21 insect cells at 72 h post inoculation with titre-normalised viral supernatants. (**B**) Live-cell imaging, scalebar = 50 µm. (**C**) polH EYFP expression readout; violin plot of plate reading measurement (plate scanning, 25 readings). Adj. *p*-value (** ≤ 0.01, *** ≤ 0.001, **** ≤ 0.0001); all samples were compared against non-VSV-G BVs; ANOVA test, *n* = 25 measurements. (**D**) Representative western blot of VSV-G and GP64 in insect cells. Actin B was used as loading control. VSV-G levels, protein extracts of cells inoculated with polH VSV-G BVs (lane 1) were additionally loaded 1:10 (lane 4). (**E**) Quantification of VSV-G expression levels, relative to (**D**). Averages + S.D. of 2 independent experiments.

To generate VSV-G-pseudotyped BVs, these alternative promoters were cloned upstream of the VSV-G coding sequence (CDS) in the pIDC plasmid [6,7] as depicted in Figure 1A. A pIDC construct containing a polH-driven VSV-G expression cassette was used as control. The pIDC plasmids were subsequently assembled via CRE-LoxP recombination in vitro with pACEMam1 [3], equipped with an EGFP CDS under the control of the ubiquitous mammalian-active CMV promoter. Assembled pACEMam1-pIDC fusions containing the expression cassettes of choice were then shuttled into DH10EMBacY bacterial cells, carrying an engineered baculoviral genome (EMBacY) in form of a bacmid, with an EYFP marker under the control of polH in the viral backbone [7] (Figure 1A). Control BVs without VSV-G pseudotyping were generated by omitting the CRE-LoxP step. The resulting BVs could then be monitored efficiently in both insect cells (by tracking EYFP) and mammalian cells (by tracking EGFP). 

The pH of insect cell culture media is mildly acidic (pH 6.0–6.5). At this pH, VSV-G is mostly found in its post-fusion conformation [22]. Combined with the hyperexpression driven by the polH promoter, this triggers insect cell membrane fusion, leading to the appearance of large syncytia [23,26]. At 72 h post-transfection in insect cells, syncytia formation could be detected when VSV-G was expressed under the control of polH, gp64, Orf-13, and Orf-81 promoters (Figure S1A), but not when expression occurred under the control of the vp39 or Lef8 promoters. Viral supernatants from transfected cells (V0) were harvested at 72 h post-transfection, and the baculoviral genome copy numbers were determined via qPCR. To eliminate the bias of variable transfection efficiencies in the assessment of VSV-G induced phenotypes, V1 amplification was started by inoculating insect cells at a multiplicity of infection (MOI) of 10 viral genome copies per insect cell (gc/cell). 72 h post-infection, insect cells were monitored for EYFP production and syncytia formation. Under standardised infection conditions, VSV-G-induced syncytia were observed only in polH VSV-G BV, suggesting that all promoters displayed substantially reduced VSV-G levels as compared to hyperactive polH (Figure 1B). Interestingly, all VSV-G BVs, independently from the promoter used, displayed reduced EYFP expression levels as compared to non-pseudotyped BVs (Figure 1C), suggesting either cell expression burden stemming from VSV-G production, VSV-G induced cytotoxicity, or both. We confirmed reduced VSV-G expression levels using the alternative promoters by western blot of cell extracts, noting that VSV-G levels from gp64, vp39, and Lef8 promoters fell below the detection limit (Figure 1D). The expression levels of Orf-81 and Orf-13 promoters, in contrast, were detectable and amounted to less than 5% of polH promoter driven expression (Figure 1E). Similar results were confirmed in western blots of concentrated BV samples (Figure S1B–D). In addition to Orf-81- and Orf-13-promoter-driven expression, VSV-G expression from the gp64 promoter was detectable confirming the presence of VSV-G on the virions (Figure S1B,D). VSV-G incorporation on BVs appeared to result in a reduced gp64 occupancy level, which may suggest a competition between different glycoproteins for the available surface of the baculoviral envelope (Figure S1C). Coomassie staining on budded baculoviruses further confirmed that GP64 levels were noticeably reduced in polH VSV-G BV, while other viral proteins were unaffected (Figure S1E). Together, these data demonstrate that alternative viral promoter usage can dramatically reduce the amount of VSV-G protein expression and incorporation on budded baculoviral particles.

### 3.2. Transduction Efficiencies of New VSV-G-Pseudotyped BVs in Mammalian Cells

We speculated that the hyperexpression of VSV-G may be unnecessary; however, no data were available about the minimum levels of VSV-G required to significantly improve BV transduction properties in mammalian cells as compared to non-pseudotyped BVs. All the promoters tested produced markedly low VSV-G expression and pseudotyping levels as compared to polH, with some even below detection levels in western blots (Figure 1). Therefore, we proceeded to characterise our new VSV-G BVs on human cells (HEK293T), in which the transduction rates by BV, as in the case of other mammalian cell lines, are significantly improved by VSV-G pseudotyping.

**Figure 2:**
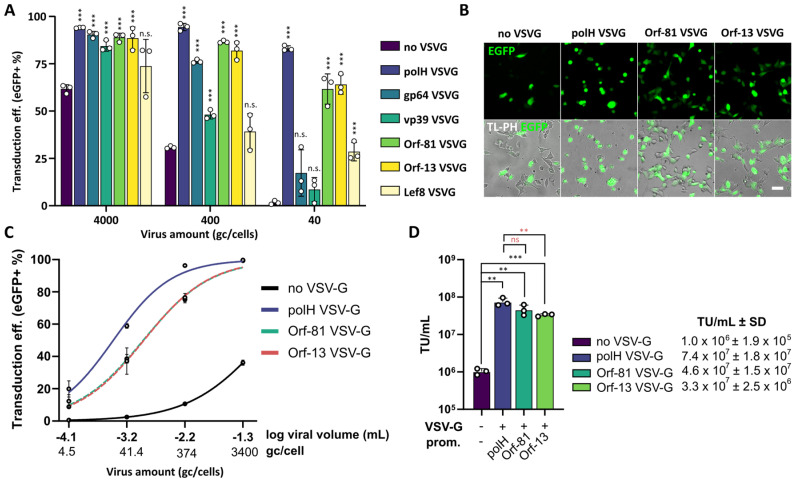
VSV-G hyperexpression is not required for efficient transduction of mammalian cells. (**A**) HEK293T transduction rates 24 h after transduction with control BVs (non-VSV-G) or BVs pseudotyped with different levels of VSV-G using alternative viral promoters. CMV EGFP expression was used as transduction marker. Mean + S.D. of 3 independent replicates. *p*-value(*** ≤ 0.001); Student’s *t*-test, all samples compared to non-VSV-G virus. (**B**) HEK293T transduced with the indicated BVs at 4000 gc/cell, scalebar = 50 µm. (**C**,**D**) Fluorescent titration assay in HEK293T at 24 h post-transduction with the indicated BVs. (**C**) Flow cytometry data of *n* = 3 independent replicates and transducing units per mL (**D**). *p*-value (** ≤ 0.01, *** ≤ 0.001), Student’s *t*-test, all samples compared to non-VSV-G virus (black symbols), Orf-13, and Orf-81 compared to polH VSV-G virus (red symbols).

Taking into account the expected differences in transduction properties conferred by the differential VSV-G pseudotyping, viral supernatants were normalised by genome copies (gc) and serially diluted to transduce target cells. Despite the low VSV-G pseudotyping levels (Figure S1B,D), all viruses tested except Lef8 outperformed non-VSV-G-pseudotyped BVs when used at a high MOI (4000 gc/cell) and produced similar transduction levels to polH VSV-G BVs (Figure 2A). Indeed, transduction efficiencies correlated well with the observed VSV-G expression and pseudotyping levels (Figure 1D and Figure S1B). A statistically significant transduction advantage conferred by VSV-G pseudotyping was maintained for promoters gp64, vp39, Orf-81, and Orf-13 (at 400 gc/cell) and only for Orf-81, Orf-13, and Lef8 promoters at higher viral dilutions (40 gc/cell) (Figure 2A,B).

Additionally, at high MOIs (4000 gc/cell), polH VSV-G BVs appeared to induce morphological changes in target cells which were not observed with Orf-81 VSV-G BVs and Orf-13 VSV-G BVs (Figure 2B). Evidently, the drastic reduction in VSV-G pseudotyping levels (<5%) using Orf-81 and Orf-13 promoters (Figure 1D and Figure S1B) did not dramatically compromise transduction efficiency in mammalian cells. Indeed, superior transduction properties were preserved over a wide range of MOIs, without a major loss in transduction efficiency when compared to polH VSV-G BV, which on average was not more than twice as effective (Figure 2C,D).

### 3.3. VSV-G Hyperexpression Affects Baculovirus Structure and Stability 

Budded baculoviral particles adopt a rod-shaped nucleocapsid surrounded by a lipid bilayer envelope. Using electron microscopy (EM), VSV-G pseudotyping using the polH promoter was initially found to profoundly affect baculovirion structures, resulting in enlarged envelopes [20], although no differences were found in later studies [23,38]. More recently, finer details of wildtype AcMNPVs were observed using electron cryo-microscopy (cryo-EM), revealing oval-shaped envelopes that are easily destroyed or lost in EM sample preparation [39]. To date, however, VSV-G BVs have not been characterised by using cryo-EM. 

**Figure 3:**
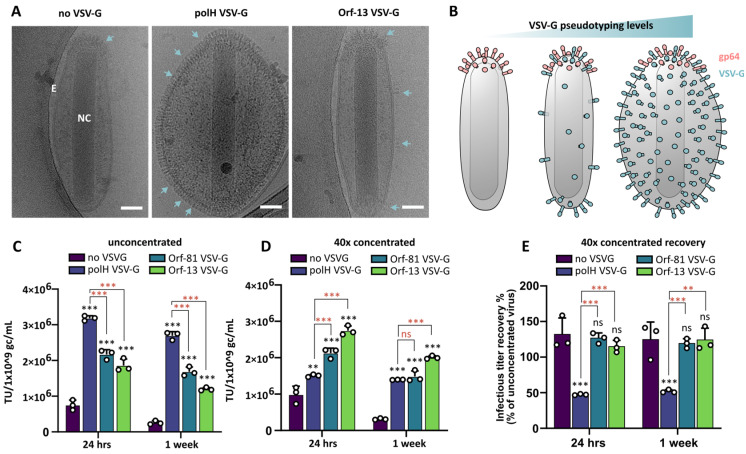
VSV-G hyperexpression affects BV morphology and stability. (**A**) Nanostructure of control baculoviruses (non-VSV-G) and VSV-G-pseudotyped BVs with standard (polH) or reduced (Orf-13) expression levels. Cyro-EM imaging, scalebar = 40 nm. E = envelope; NC = nucleocapsid. Arrows indicate position of glycoproteins (GP64 or VSV-G) embedded in the lipid bilayer envelope. (**B**) Model of BVs nanostructures with increasing VSV-G incorporation levels. (**C**–**E**) Transducing viral titres upon concentration and resuspension at 1 (**C**) or 1:40 (**D**) of the original volume assessed on HEK293T via flow cytometry; viral stocks were tested after 24 h or 1 week storage at 4 °C. (**D**) Transducing titres recovery of 40× concentrated viral stocks compared to their unconcentrated controls. Mean + S.D. of 3 independent replicates (**C**–**E**). *p*-value (** ≤ 0.01, *** ≤ 0.001), Student’s t-test, all samples compared to non-VSV-G virus (black symbols), Orf-81 and Orf-13 compared to polH VSV-G virus (red symbols).

To elucidate the impact of VSV-G hyperexpression and pseudotyping on BV nanostructures, we performed cryo-EM imaging of BVs with standard or reduced VSV-G pseudotyping levels and compared them to non-VSV-G BVs. Comparison of BVs with intact envelopes clearly revealed the pronounced effect of excessive VSV-G levels on BV morphology (Figure 3A,B), resulting in significantly enlarged envelopes. As previously observed [23], BVs with normal envelopes could also be found in polH VSV-G viruses, although they displayed remarkably lower levels of VSV-G presumably due to early budding, before polH-driven peak expression. Since GP64 typically occupies only the apex of the BV envelope [39], increased protein incorporation within the lipid bilayer was interpreted as VSV-G occupancy. In Orf-13 VSV-G BVs, the appearance of the envelope was overall similar to non-pseudotyped control BVs (non-VSV-G) (Figure 3A,B). As for polH VSV-G BVs, Orf-13 VSV-G also displayed a certain degree of variation in VSV-G incorporation levels. In the latter virus, however, the highest VSV-G incorporation levels remained well below polH VSV-G BVs and did not appear to cause any morphological alterations (Figure S2A). Alongside structurally intact BVs with oval-shaped envelopes, we also observed BVs with loosely attached envelopes, naked nucleocapsids and vesicles as previously described [39] (Figure S2). Vesiculation has recently been observed for wildtype BVs [40] and appeared to be more severe in polH VSV-G BVs as previously reported [23]. It remains unclear whether these vesicles have any significance or whether their appearance, consistent with the presence of naked nucleocapsids, may be an artifact of sample preparation (e.g., virus concentration).

In our experience, polH VSV-G BV titres tend to dramatically decrease after the concentration and storage of viral supernatants, in contrast to standard BVs which can be concentrated and stored in a range of conditions without excessive loss of infectious viral titre [41]. We speculated that the defects in the BV nanostructure caused by VSV-G hyperexpression could influence viral stability upon concentration either by enhancing viral particle aggregation [42] or by triggering membrane envelope instability as suggested by cryo-EM imaging. To analyse the impact of VSV-G levels on BV transduction properties, we concentrated titre-normalised (1 × 10^9^ gc/mL) viral supernatants using high-speed centrifugation and resuspended the viral pellets in either 1 (unconcentrated) or 1:40 (40-fold concentration) volumes of DPBS pH 7.4. Transducing titres in mammalian cells (HEK293T) were evaluated via flow cytometry (as in Figure 2C) after 24 h or 1 week of viral storage at 4 °C. The relative transduction efficiencies of unconcentrated virus remained stable (Figure 3C) and consistent with previous data (Figure 2C,D). Upon high concentration, however, polH VSV-G BVs titres were dramatically affected (Figure 3D), while Orf-81 VSV-G BV and Orf-13 VSV-G BV titres remained unaffected. The storage of concentrated viruses for up to 1 week in the absence of any additives did not dramatically affect viral titres either (Figure 3C,D), confirming that high-concentration storage conditions, but not storage time (Figure S2B), were responsible for polH VSV-G BV titre loss. Indeed, when compared to their respective unconcentrated stocks, 40× concentrated polH VSV-G BVs lost up to 50% of transduction titre, while titres were unaffected for control viruses (non-VSV-G), Orf-81 VSV-G BVs, and Orf-13 VSV-G BVs (Figure 3E). 

In summary, these data show that VSV-G hyperexpression under the control of the polH promoter alters BV envelope nanostructures, which in turn are responsible for increased viral instability, notably under high-concentration storage conditions.

### 3.4. BVs Pseudotyped with Reduced VSV-G Levels Exhibit Reduced Toxicity in Immortalised Cells and Primary HUVEC

**Figure 4:**
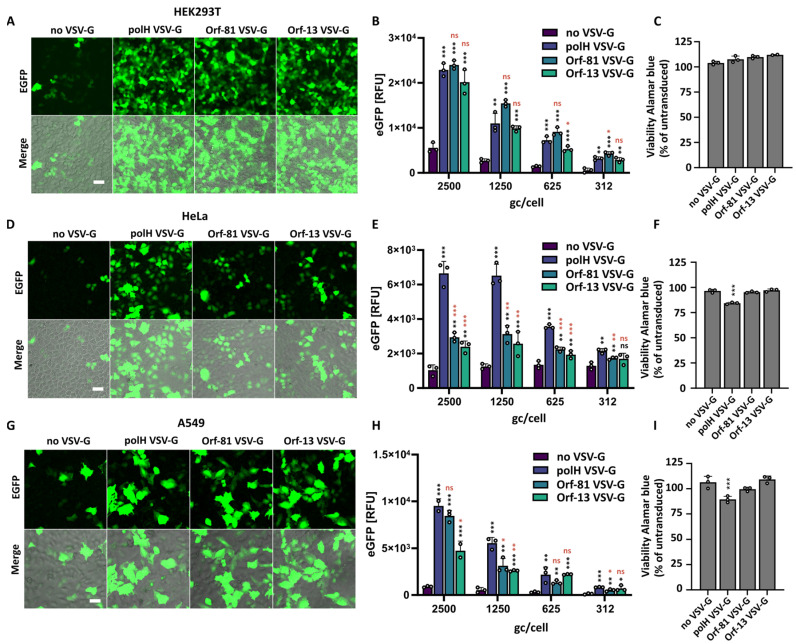
Analysis of transgene expression and viability in immortalised human cell lines. HEK293T (**A**–**C**), HeLa (**D**–**F**), and A549 (**G**–**I**). (**A**,**D**,**G**) Live brightfield imaging of cells transduced with the indicated BVs at 2500 gc/cell. Scalebar = 50 μm. (**B**,**E**,**H**) Transgene (EGFP) expression levels in cells transduced with serial dilutions of polH, Orf-81, and Orf-13 VSV-G BVs or with a non-pseudotyped control virus (non-VSV-G). Plate reader, EGFP relative fluorescence units (RFUs), mean ± S.D. *n* = 3 independent replicates, *p*-value (* ≤ 0.05, ** ≤ 0.01, *** ≤ 0.001), Student’s *t*-test, all samples compared to non-VSV-G virus (black symbols), Orf-81 and Orf-13 compared to polH VSV-G virus (red symbols). (**C**,**F**,**I**) Viability of transduced cells measured via alamarBlue staining at 48 h post-transduction (2500 gc/cell). alamarBlue RFUs are presented as % of the untransduced control. Mean + S.D of *n* = 3 independent replicates. *p*-value (*** ≤ 0.001), Student’s *t*-test, all samples compared to non-VSV-G virus.

**Figure 5:**
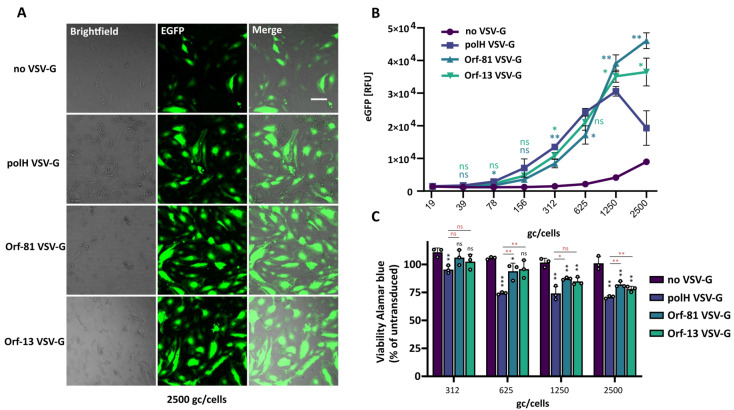
Analysis of transgene expression and viability in primary HUVEC. (**A**) Live brightfield imaging of HUVEC transduced with the indicated BVs. Scalebar = 50 μm. (**B**) Transgene (EGFP) expression dynamics in HUVEC transduced with serial dilutions of polH, Orf-81, and Orf-13 VSV-G BVs or with a non-pseudotyped control virus (no VSV). Plate reader, EGFP relative fluorescence units (RFUs), mean ± S.D. *n* = 3 independent replicates. *p*-value (* ≤ 0.05, ** ≤ 0.01), Student’s *t*-test, Orf-13 (light green symbols) and Orf-81 (dark green symbols) samples compared to polH VSV-G virus. (**C**) Viability of transduced cells measured via alamarBlue staining. alamarBlue RFUs are presented as % of the untransduced control. Mean + S.D of *n* = 3 independent replicates. *p*-value (* ≤ 0.05, ** ≤ 0.01, *** ≤ 0.001), Student’s *t*-test, all samples compared to non-VSV-G virus (black symbols), Orf-81 and Orf-13 compared to polH VSV-G virus (red symbols).

Our results demonstrated that significantly reduced VSV-G pseudotyping levels suffice for achieving similar transduction levels in HEK293T when compared to polH VSV-G BVs. Nonetheless, it is important to assess whether these reduced levels are sufficient for ensuring high transduction rates in other cell lines, including primary cells which are usually more susceptible to stresses, including transduction. While the cytotoxicity of polH VSV-G BVs is considered to be low, we have previously observed cell-type-dependent decreases in cell viability at high MOIs at 48 h post-transduction [16], particularly concerning primary cells. We thus compared EGFP expression levels and cell viability in immortalised human cells (HEK293T, HeLa, and A549) (Figure 4) and primary human umbilical vein endothelial cells (HUVEC) (Figure 5) transduced with polH, Orf-81, and Orf-13 VSV-G BVs. Titres were normalised by qPCR, and cells were transduced with serially diluted viruses starting from 2.5 × 10^3^ gc/cell, corresponding to 100 transducing units per cell (TU/cell) for the polH VSV-G virus, which is within the standard range used in the literature. 

In HEK293T, Orf-81 and Orf-13 VSV-G BVs displayed transduction rates in the range of polH VSV-G BV, with matching transgene expression levels and an absence of defects in viability at 48 h post-transduction (Figure 4A–C). In HeLa, when compared to non-pseudotyped (wt) BV, Orf-81 and Orf-13 VSV-G BVs resulted in higher transgene expression efficiencies at all the tested dilutions (Figure 4D). BVs with reduced levels of VSV-G, however, displayed a 2- to 3-fold decrease in transgene expression rates when compared to polH VSV-G BVs (Figure 4E). A reduction in cell viability was observed for polH VSV-G BV, but not for Orf-81 VSV-G BVs and Orf-13 VSV-G BVs (Figure 4F). 

In A549, reduced VSV-G levels in BVs increased transduction and transgene expression levels, compared to those of non-pseudotyped BVs, to levels close to polH VSV-G BV and in the absence of any negative effects on cell viability (Figure 4G–I). Compared to non-pseudotyped (wt) BVs, transduction with Orf-81 and Orf-13 VSV-G BVs resulted in higher transgene expression efficiencies at all the tested dilutions, except for Orf-13 at 312 gc/cell (Figure 4H).

In HUVEC, all VSV-G-pseudotyped BVs, notwithstanding the promoter used, displayed higher EGFP expression levels than their non-pseudotyped counterparts (Figure 5A,B). At the highest viral titre used in these experiments, HUVEC transduced with polH VSV-G BVs displayed marked morphological changes at 48 h post-transduction, which were substantially less pronounced in cells transduced with Orf-81 and Orf-13 VSV-G BVs (Figure 5A). Compared to polH VSV-G, Orf-13 and Orf-81 VSV-G BVs displayed slightly lower transgene expression, at up to 625 gc/cell (Figure 5B). At high viral doses, EGFP expression was reduced for polH VSV-G BVs, while it kept increasing and reaching a plateau for Orf-81 and Orf-13 VSV-G BVs (Figure 5B). Reduced transgene expression at high MOIs appeared to be linked to a dose-dependent decrease in cell viability, which was less apparent with Orf-81 and Orf-13 VSV-G BVs (Figure 5C), with a concomitant increase in EGFP expression levels.

These data highlight that high transduction and transgene expression levels can be achieved in mammalian cells using BVs with significantly reduced VSV-G pseudotyping levels (<5% of the level in polH VSV-G BV). In all the tested cell lines, Orf-81 and Orf-81 VSV-G BVs achieved higher transgene expression levels than non-pseudotyped BVs. Some cell lines, however, appear to still benefit from higher VSV-G levels (e.g., HeLa), highlighting that additional finetuning of VSV-G expression levels might be beneficial in some instances. More importantly, BVs with reduced VSV-G levels mitigated cytotoxicity in primary HUVEC cells, partially rescuing viability defects and contributing to higher transgene expression levels at high MOTs as compared to polH VSV-G BVs.

### 3.5. Reduced VSV-G Pseudotyping Supports Efficient CRISPR-Mediated Gene Editing Using All-in-One BVs

Due to their exceptionally large DNA cargo capacity, BVs represent particularly promising tools for multigene delivery in mammalian cells. We and others have successfully delivered complex CRISPR-based editing tools using single all-in-one BVs [3,13,14,15,16,29]. In all cases, polH VSV-G BVs were employed to ensure high transduction rates and editing efficiencies. 

**Figure 6:**
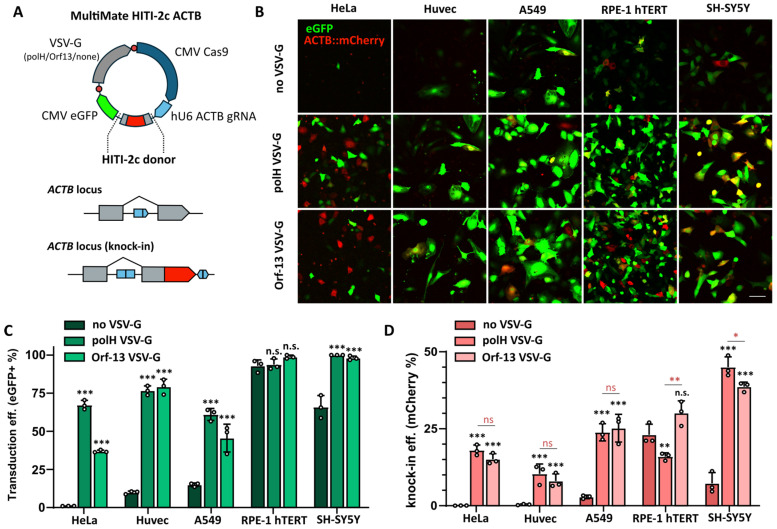
Assessment of the impact of reduced VSV-G pseudotyping levels on gene editing efficiencies of all-in-one CRISPR BVs. (**A**) Schematic representation of MultiMate HITI-2c ACTB all-in-one vector encoding Cas9, gRNA, and HITI-2c donor. To generate VSV-G-pseudotyped BVs, pIDC vectors were incorporated into MultiMate HITI-2c ACTB via in vitro CRE-mediated recombination. Upon successful knock-in, endogenous ACTB is fused to a C-terminal mCherry fluorescence marker. (**B**–**D**) Analysis of EGFP (transduction efficiency) and mCherry (knock-in) efficiency in the indicated cell lines at 48 h post-transduction with control (non-VSV-G), polH VSV-G, and Orf-13 VSV-G BVs encoding the MultiMate HITI-2c ACTB construct depicted in (**A**). (**B**) Confocal live-cell imaging, scalebar = 100 μm; (**C**) transduction and (**D**) knock-in efficiencies at 48 h post-transduction. Histogram of flow cytometry data, mean + S.D of *n* = 3 independent replicates. *p*-value (* ≤ 0.05, ** ≤ 0.01, *** ≤ 0.001), Student’s *t*-test, all samples compared to non-VSV-G virus (black symbols), Orf-13 compared to polH VSV-G virus (red symbols).

Here, we sought to evaluate gene editing efficiencies elicited by all-in-one BVs pseudotyped with reduced VSV-G levels and compare them to either control BVs (non-VSV-G) or standard polH VSV-G BVs. The all-in-one BV chosen was previously engineered to simultaneously deliver Cas9, gRNA, and a homology-independent targeted integration (HITI-2c) donor for β-ACTIN (ACTB) C-terminal tagging with a fluorescent mCherry protein (Figure 6A). The BV was additionally equipped with a CMV EGFP expression cassette, to monitor transduction efficiency (EGFP) and knock-in efficiency (mCherry) as previously described [15]. In addition to HeLa, HUVEC, and A549, retinal pigmented epithelial cells (RPE-1 hTERT) and neuroblastoma cells (SH-SY5Y) were transduced at 2500 gc/cell and analysed by microscopy and flow cytometry (Figure 6B–D). At 48 h post-transduction, the appearance of mCherry+ cells with a distinct actin subcellular localisation indicated successful gene-editing events (Figure 6B) as previously reported [15]. The transduction and editing efficiencies of Orf-13 VSV-G BVs were overall similar to polH VSV-G BV and higher than the control (non-VSV-G BV) (Figure 6B–D). The use of non-pseudotyped (non-VSV-G) BVs, in contrast, did not result in gene editing in HeLa, HUVEC, A549, and SH-SY5Y (Figure 6D).

One notable exception was RPE-1 hTERT, which was efficiently transduced by all BVs regardless of the presence or absence of VSV-G (Figure 6C). In RPE-1 hTERT, however, polH VSV-G BV transduction resulted in significantly lower gene editing efficiencies than both non-pseudotyped BVs and Orf-13 VSV-G BVs (Figure 6D). In HeLa, while transduction efficiencies of Orf-13 VSV-G BVs were lower than those of polH VSV-G BVs (Figure 6C), their gene editing efficiencies were unaffected (Figure 6D). In SH-SY5Y, Orf-13 VSV-G BV transduction resulted in slightly lower gene editing efficiencies (38.6 ± 1.5%) than those of polH VSV-G (45 ± 3.3%). Taken together, these data demonstrate that reduced VSV-G pseudotyping levels are entirely sufficient for efficient gene editing by all-in-one CRISPR BVs and do not impair gene editing efficiencies in a range of human cell lines, including primary HUVEC. 

## 4. Discussion

Essentially, all currently existing data and observations with VSV-G-pseudotyped BVs rely on VSV-G expression driven by the hyperactive polH very late viral promoter, which is the strongest promoter known to be active in the baculovirus infectious cycle. We asked whether the detrimental effects observed, notably the cytotoxicity and the titre loss during the storage of concentrated virus, could be alleviated by providing lower amounts of VSV-G decorating the envelope of the pseudotyped baculovirions. 

In this study, we addressed this by tuning the VSV-G level, using a range of promoters (gp64, vp39, Orf-13, Orf-81, and Lef8) with varying strengths based on transcriptomic data [32] and characterising the properties of the resulting differentially VSV-G-pseudotyped BVs regarding the abovementioned key properties. With the exception of the gp64 promoter, which is active at early and late stages during infection, all the selected promoters were chosen from genes expressed during the late stage. Based on previously published transcriptomic data [32], candidate promoters were chosen to have varying degrees of expression in insect cells and little-to-no leaky expression in transduced mammalian cells.

The expressions from most of the promoters tested resulted in surprisingly low levels of VSV-G, with some below detection levels including well-characterised promoters vp39 and gp64 [30,31]. Nevertheless, expression of VSV-G from Orf-81 and Orf-13 promoters, amounting to less than 5% of polH-driven VSV-G, sufficed for approximating the transduction efficiencies obtained when using polH VSV-G BVs, confirming our hypothesis that VSV-G hyperexpression is indeed not required for efficient transduction across a range of different mammalian cell lines. 

We confirmed by cryo-EM that VSV-G hyperexpression has dramatic effects on the envelope structure of budded virions, contributing to a significant enlargement of viral envelopes, suggesting increased membrane tension and fragility. These results contribute to elucidate VSV-G BV nanostructures, additionally resolving an apparent contradiction between previous studies [20,23]. As could be expected, our Orf-13 VSV-G with significantly (<5%) reduced VSV-G pseudotyping levels displayed envelope shapes very similar to non-pseudotyped BV, while retaining high transduction efficiencies in mammalian cells. 

Although we—and presumably others as well—have routinely experienced it, polH VSV-G BV instability at high concentrations has not been well documented previously. We believe that either an increase in membrane fragility or accelerated formation of viral aggregates [42] could be responsible for this decrease in transducing titre recovery.

In marked contrast, our BVs with reduced VSV-G pseudotyping levels maintained their titres when being concentrated, similar to non-pseudotyped BVs. Our data show that after concentration, up to 50% of polH VSV-G BV titre is lost, presumably due to the presence of aggregates or naked nucleocapsids which can still significantly contribute to toxicity in target cells. From a manufacturing perspective, concentration is a critical step as for most applications in the gene therapy space, storage of high-titre viral stock is a key prerequisite regardless of upstream purification strategies [18,43]. 

We showed that significantly reduced VSV-G pseudotyping levels did not dramatically affect transduction efficiencies of most target mammalian cells. While transduction rates from Orf-81 and Orf-13 VSV-G BVs were approximately 2 to 3 folds lower than polH VSV-G BVs in most cell types, some cell types (e.g., HeLa) were transduced at substantially higher levels in the presence of polH VSV-G, highlighting that higher VSV-G levels might be essential in some instances. We also observed that reduced VSV-G levels could reduce cell toxicity at high MOTs in primary cells. In this context, Orf-13 and Orf-81 VSV-G BVs supported higher transgene expression levels than polH VSV-G at high MOTs, partially, albeit not completely, rescuing VSV-G induced cytotoxicity. Notably, our new BVs supported the efficient delivery of complex CRISPR multigene constructs, with transduction and gene editing efficiencies overall comparable to what we achieved with polH VSV-G BVs. In this context, however, gene editing efficiencies remained highly cell-type-dependent, underlining the importance that other factors, such as the activities of DNA-repair pathways, play on the overall gene-editing outcome. 

In conclusion, we have demonstrated that VSV-G pseudotyping levels can be significantly reduced to less than 5% of the levels present in currently employed VSV-G-pseudotyped BVs, without excessively compromising transduction efficiencies and with little-to-no impairment of gene editing efficiencies, in a range of mammalian cell lines including primary cells. At the same time, we showed that by reducing VSV-G levels, previously observed drawbacks caused by excessive VSV-G pseudotyping were significantly alleviated. The benefits of VSV-G level reduction include reduced cytotoxicity in target cells, improved viral nanostructures, and superior stability of concentrated viral stocks. While in the future additional promoters with slightly increased expression levels could be helpful for fully matching polH VSV-G transduction efficiencies, we anticipate that the Orf-13 VSV-G and Orf-81 VSV-G BVs we generated and characterised here may thus be suitable alternatives for current VSV-G-pseudotyped BVs, expanding the range and scope of BV applications in mammalian cells, including multigene delivery, gene editing, and gene therapy approaches. 

## Data Availability

All plasmids and recombinant bacmids generated in this study are available upon reasonable request. The promoter sequences used in this study are provided in Supplementary Table S1. The plasmid sequences are available in Table S2.

## Supplementary Materials

The following supporting information can be downloaded at https://www.mdpi.com/article/10.3390/v16091475/s1, Table S1: Sequence, position, and length of baculovirus promoters used in this study; Figure S1: (Relative to Figure 1); Figure S2: (Relative to Figure 3), Table S2: Plasmid sequences and information.
