# Supplementary material for: Tuning VSV-G Expression Improves Baculovirus Integrity, Stability and Mammalian Cell Transduction Efficiency
Source: Viruses. 2024 Sep 17;16(9):1475. doi: 10.3390/v16091475 (PMC11437408; doi:10.3390/v16091475)
Supplement: Supplementary file 1 [file viruses-16-01475-s001.zip › viruses-3203687-supplementary.pdf]

Table S1. Sequence, position and length of baculovirus promoters used in this study

promoter	Position[1]	Sequence	Length (bp)
polH	-92/-1	atcatggagataattaaaatgataaccatctcgcaaataaataagtattttactgttttcgtaacagtttt gtaataaaaaaacctataaat	92
gp64	-152/+54*	tgtcgactgagcgtccgtgttcatgatcccgtttttataacagccagataaaaataatcttatcaatta agataaaaagataagattattaatctaacaacgtgccttgtgtcacgtaggccagataacggtcgg gtatataagatgcctcaatgctactagtaaatcagtcacaccaaggcttcaataaggaacacacaa gcaag	206
vp39	-329/-1	gtcttgtaaggcagtttgatttctttgctttctctccacaccaacggcaccaacgcgttggtatcttta ggccaataaacaaatttttgtgtttggaattagtctttttcacgcttgatattatgttattgcaagcgct ctgaataggtatacgagtgcgaaagccgttttcgtcgtacaaatcgaaatattgttgtgccagcgaa taattaggaacaatataagaatttaaaattttatacaacaaatcttggctaaaatttattgaataagaga tttctttctcaatcacaaaatcgccgtagtccatatttataacggcaacaat	329
Orf-13	-474/-1	ggtcgtagacgccgattacaaaaattatgttgacgaacacgatttaatgctgaaaatttacattggc gccacggcgtttctgttgttttacacggaagagaacgtgtcaagagtcatgtataccggcaaccgt ggatttcatttgtggttaaaattcaccgacaagtttaaaatcacgtccgctcaaaatgttcgcgtgcat cggtacaaggctttcgagaaacctgcaaagttggacagtgattacattcagccgggcagttttgc gcattgtgtgagagaggccgtacgtttgtacgttccgcatatgcaagattcaaacttggacgcgct cacgttgcagtattggccggacgtggacagggatatttttgtaacgttaacaaacaaatacgcgc accgtacagctataattataagggaacgaaattttctcgttgtataacaaaggaattgctagacaag ctaaagca	474
Orf-81	-175/-39*	ataatgtttaactgcgccgaaataaagttgaacaagggccaaatgttatttcaaacaaaaatttggc ggtccgataatagtaagacggacgctgctgtacacacgtcgtcgcccaaacgcactgtagaaac cgcga	136
Lef8	-500/-1	aaaaatattgaccaaagcttttctgagtttttgtatttcttcatttacagattcgccaatttgtaaaaaga ccgtaatcaatggatcgtacgattccaagtctgtattttttttcaattttaacacatcgtcatagtattgct ttttgacactattactaagcactaaacgcgcatttttttattaaaatcaacacgttacgcaccgtgttgta atggtgcttttcgtcgatactgttatacggtctaacttgtttcgtcaccacgttggccattctgtacact gttaaaataaaatcgttgtcaaacttttggtccggaacgtattgcatttttaaacattgataataatcaa tttcttccaagttgcaaaaccccaaaaaagattcatttttggcatgacagtgttcgttttgtcgaatgc gtttggccgtttcatccgaaacaaccgaccgttcacgaacacgtttagacataattagtgcgcagta atcgcttcacg	500

[1] Positions are relative to the ATG of their respective CDS on wild-type AcMNPV E2 genome (GenBank KM667940). * 3′ terminus of gp64 promoter (+54) and Orf-13 promoter (-39) is due to variations in CDS annotations in KM667940.

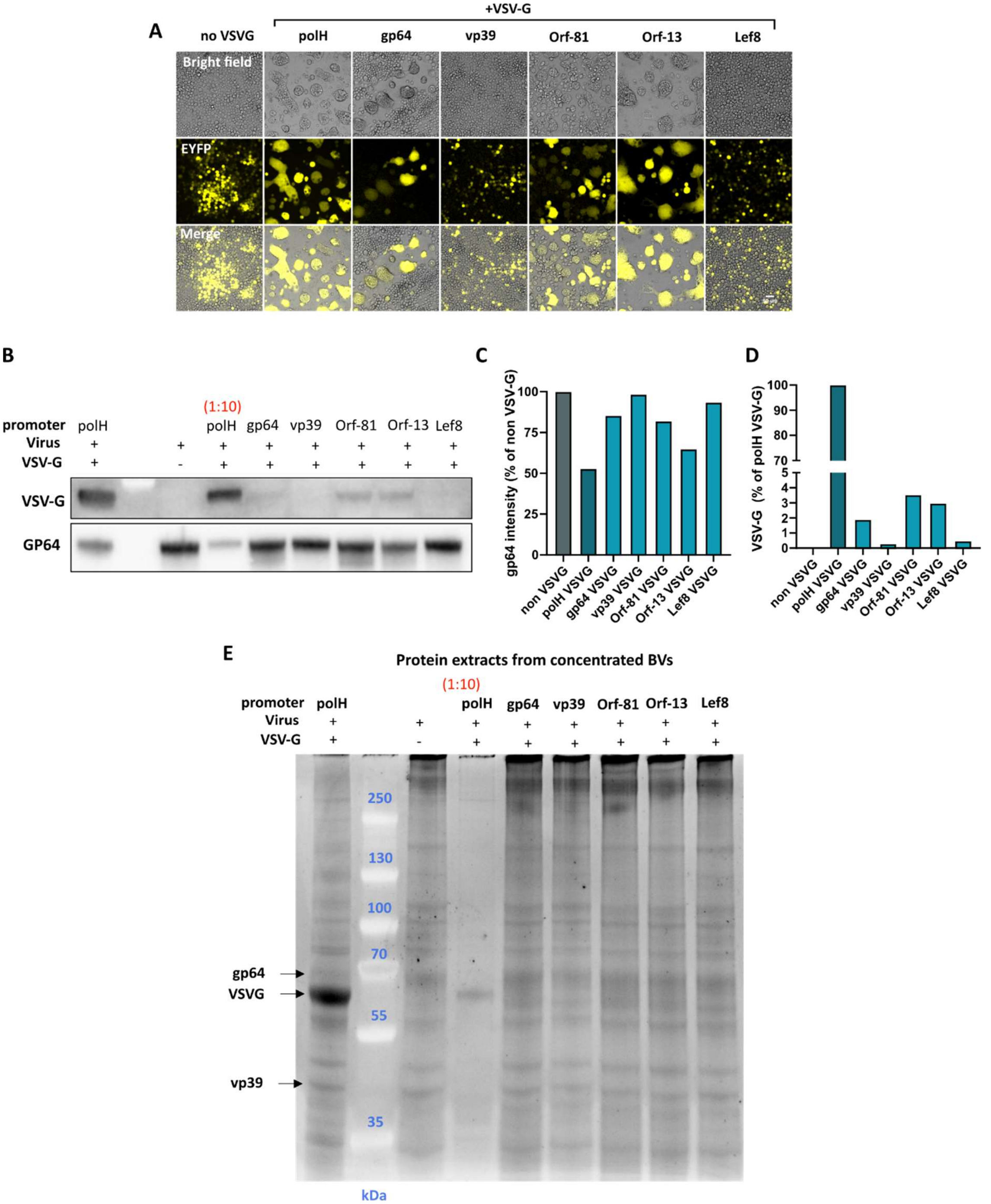

Supplementary Figure S1. (Relative to Figure 1) - Baculovirus VSV-G pseudotyping using alternative viral promoters. A) Live cell imaging of Sf21 insect cells at 72 hours post transfection with the indicated bacmids, scalebar = 50 µm. B-D) Western blot (B) and relative quantification (C,D) of gp64 and VSV-G on concentrated, and titer normalized, budded virions. D) Coomassie staining of whole protein extracts from concentrated, titer normalized, BVs. Molecular weights are added highlighted in blue (kDa). In C and E protein extracts of polH VSV-G BVs (lane 1) were additionally loaded 1:10 (lane 4)

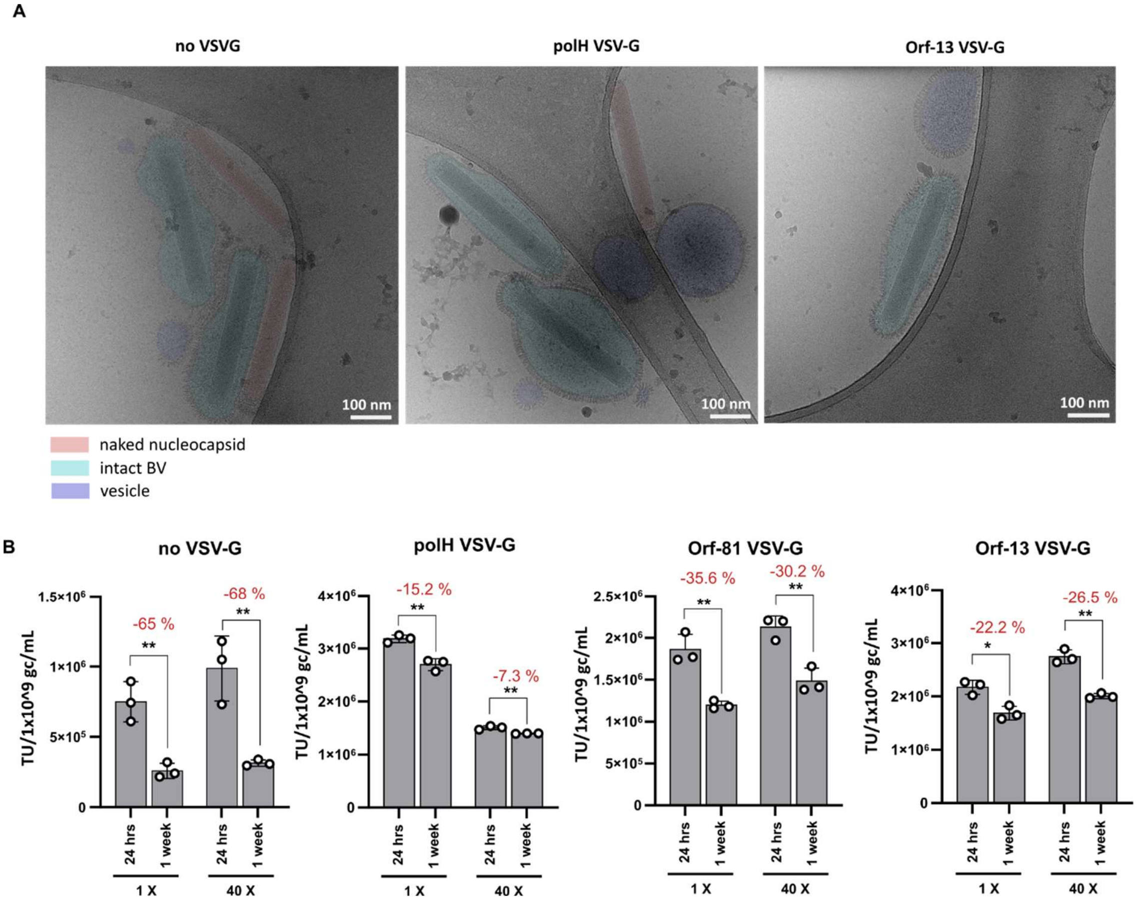

Supplementary Figure S2. (Relative to Figure 3) – A) Additional BVs nanostructures identified in cryo-EM imaging for the indicated BVs. Coloring is added to aid identification of intact BVs (green), vesicles (blue) and naked nucleocapsids (pink). Scalebar is 100 nm. B) Comparison of viral titer loss depending on time of storage. 1x = unconcentrated virus; 40 x = viral stocks concentrated in 1:40 of the original volume. Data re-analyzed from Figure 3C and D. Mean + S.D. of 3 independent replicates. P Value (*= <0.05, **=<0.01, ***=<0.001), Student's t-test. Percentage losses in viral titer between 24 hours and 1 week storage time are indicated in red.
